# Differential effects of bilingualism and culture on early attention: a longitudinal study in the U.S., Argentina, and Vietnam

**DOI:** 10.3389/fpsyg.2015.00795

**Published:** 2015-06-18

**Authors:** Crystal D. Tran, Maria M. Arredondo, Hanako Yoshida

**Affiliations:** ^1^Department of Psychology, University of HoustonHouston, TX, USA; ^2^Department of Psychology, University of MichiganAnn Arbor, MI, USA

**Keywords:** attentional control, Attention Network Test, bilingual, cross-cultural comparison, attentional processes, bilingual advantage, longitudinal

## Abstract

A large body of literature suggests that bilingualism strongly influences attentional processes among a variety of age groups. Increasing studies, however, indicate that culture may also have measurable effects on attentional processes. Bilinguals are often exposed to multiple cultural backgrounds, therefore, it is unclear if being exposed to multiple languages and culture together influence attentional processes, or if the effect themselves are uniquely linked to different attentional processes. The present study explores the relevancy of different attentional processes—alerting, orienting, and executive control—to language and to culture. In the present study, 97 3-years-old (Mean age = 38.78 months) monolingual and bilingual children from three countries (the U.S., Argentina, and Vietnam) were longitudinally tested for a total of five time points on a commonly used non-linguistic attentional paradigm—the Attention Network Test. Results demonstrate that when other factors are controlled (e.g., socio-economic status, vocabulary knowledge, age), culture plays an important role on the development of the alerting and executive control attentional network, while language status was only significant on the executive control attentional network. The present study indicates that culture may interact with bilingualism to further explain previous reported advantages, as well as elucidate the increasing disparity surrounding cognitive advantages in bilingual literature.

## Introduction

Through exposure and constant use of multiple languages, bilingual learners are required to make fast and adaptive changes from context-to-context, and this constant shifting and controlling one’s attention to the relevant language has been found to have measureable effects on attentional control (e.g., Bialystok, 1999; Bialystok and Martin, 2004; Carlson and Meltzoff, 2008; Kovács and Mehler, 2009; Yoshida et al., 2011). This effect has been referred as part of the bilingual cognitive advantage (e.g., Kroll and Bialystok, 2013). Indeed, a large body of research has attributed positive attentional consequences through multiple language learning experiences (i.e., bilingualism) over the past decade. However, a growing body of literature has recently challenged this view, citing mixed results on the bilingual cognitive advantage when samples and outside factors such as socio-economic status (SES), age, and vocabulary knowledge are controlled (e.g., Paap and Greenberg, 2013; Anton et al., 2014; Duñabeitia et al., 2014; Gathercole et al., 2014). The present study focuses on one factor, individuals’ cultural background, to address the nature of the mixed results. Individuals’ cultural background has been linked to attentional processing and control (Oh and Lewis, 2008; Varnum et al., 2009; Yang et al., 2011; Kuwabara and Smith, 2012), yet this cultural influence has not been systematically addressed in the framework of bilingual cognitive advantage. Considering the potential cultural relevancy to the bilingual cognitive advantage is important to provide insights into the nature of the mixed results through understanding how language and culture are related in their influence on early attentional control. In this framework, the present study specifically considers Eastern cultural influences on the development of attention control by using the Attention Network Test (ANT; Fan et al., 2002).

The ANT measures general attentional skills by means of the performance of three different attentional networks—alerting, orienting, and executive control (Fan et al., 2002; Rueda et al., 2004). These networks are relevant to reading, mathematical learning, and academic achievement (Swanson and Jerman, 2006; Tannock, 2008), and in the present study, we hypothesize that these different attentional processes are *uniquely* relevant to bilingualism and to culture. The ANT has become one of the most commonly used attention measure that is devoid of language experience or metalinguistic knowledge (e.g., Fan et al., 2002; Carlson and Meltzoff, 2008; Costa et al., 2008) to assess for and make predictions of bilingual cognitive advantage in adults (Colzato et al., 2008; Costa et al., 2008, 2009; Hernandez et al., 2010; Pelham and Abrams, 2014) and children of varying ages (Yang, 2007; Carlson and Meltzoff, 2008; Yang et al., 2011; Yoshida et al., 2011; Kapa and Colombo, 2013; Yang, unpublished).

To systematically study the cultural effect in the bilingual cognitive advantage framework, the present study includes child participants who are exposed to one of the three cultures that vary on the cross-culture continuum ranging from more individualistic to more collectivistic: the U.S., Argentina, and Vietnam, respectively (Hofstede, 1980, 2001, 2003). These three cultures are chosen to represent *three distinct points* in the cultural continuum – the Western, Western-European with Latin influences, and Eastern culture. Further, the present study includes Vietnam and Argentina due to the lack of studies on the following countries when considering bilingualism and cultural influences on cognition. The inclusion of a wide range of cultures in the present study offers insights into the potential graded effects of cultural background on attentional processes.

### Cultural Implications on Early Attention

Bilinguals come from diverse environments influenced by various factors that include family and cultural values (Carlson and Meltzoff, 2008; Oh and Lewis, 2008; Bialystok and Viswanathan, 2009; Barac and Bialystok, 2012), immigration status (Romaine, 1989), linguistic background (Bialystok, 2009; Barac and Bialystok, 2012), and SES background (Calvo and Bialystok, 2009). The present focus on the role of cultural background is motivated by increasing studies suggesting how culture plays an important role in attention (e.g., Varnum et al., 2009; Yang et al., 2011; Yang, unpublished) and how individual’s cultural history shapes the way individuals notice and attend to visual cues and stimuli (Ji et al., 2000; Nisbett et al., 2001; Masuda and Nisbett, 2001, 2006; Kitayama et al., 2003; Nisbett, 2003; Nisbett and Masuda, 2003; Chua et al., 2005; Nisbett and Miyamoto, 2005; Varnum et al., 2009; Kuwabara et al., 2011; Kuwabara and Smith, 2012). In particular, an Eastern cultural advantage was found over Western cultures on children’s performance on overall attention, as measured by the ANT (Yang et al., 2011; Yang, unpublished). This advantage is often considered to be the result of child-rearing values (i.e., disciplined behavior and early behavioral regulation) specific to Eastern cultural practices (Oh and Lewis, 2008; Yang et al., 2011; Yang, unpublished). Distinctions between Eastern and Western cultures have been characterized and documented based on the structural degree of these societies as they differ on people’s goals, needs, collectivism, and individualism (Triandis, 1994, 1995). Eastern (or collectivistic) cultures place emphasis on obedience to authority figures, interdependence, early maintenance of self-regulation/impulse control, strict academic training, and less on play (e.g., Tobin et al., 1989; Ho, 1994; Wu, 1996; Chen et al., 1998; Nisbett et al., 2001; Parmar et al., 2004; Oh and Lewis, 2008). Western (or individualistic) cultures, on the other hand, value practices of individualism, independence, self-expression, and play (e.g., Ahadi et al., 1993; Chao and Tseng, 2002; Parmar et al., 2004). Indeed, recent works using different measures of attentional flexibility suggest that parental rearing and formal instructional practices appear to influence the development of attention differently among children from Eastern and Western cultures (Mezzacappa, 2004; Oh and Lewis, 2008; Yang et al., 2011; Yang, unpublished). One such study has further suggested that the *cultural effect* behaves similarly to the *bilingual advantage effect* in attention (Yang et al., 2011). In this study, a Korean advantage between two monolingual groups (Korean- and English-speaking 3.5-years-old children) was demonstrated on the ANT, suggesting the potential role of culture on general attentional control. This Korean advantage has been explained by the Eastern cultural practices/values on collectivism and parenting attitudes (e.g., Ahadi et al., 1993; Chen et al., 1998; Vinden, 2001; Chao and Tseng, 2002).

In addition to cultural advantages in attentional control, there are a number of studies reporting linkages between cultural differences in processing visual attention (e.g., Masuda and Nisbett, 2001, 2006; Nisbett et al., 2001; Nisbett, 2003; Nisbett and Miyamoto, 2005; Varnum et al., 2009; Kuwabara et al., 2011; Kuwabara and Smith, 2012). In particular, adults in Western cultures were shown to exhibit more focused attention, whereas adults in Eastern cultures demonstrated broader and more distributed attention. For instance, when adults participated in attentional and perceptual tasks, those in Western cultures (i.e., the U.S.) tend to narrowly process visual information to individual target objects that is less reliant on surrounding features, while those in Eastern cultures (i.e., Japan) broadly process information dependent on the surrounding contexts. This cross-cultural phenomenon has often been reminiscent to the individualistic and collectivistic societal structure frequently adopted in Western and Eastern cultures, respectively (Triandis, 1994, 1995; Varnum et al., 2009). Here, individualistic cultures tend to orient around the self, whereas collectivistic cultures value working in groups and are reliant to those around (Rothwell, 2010). Specific to the present hypothesis, differences in processing visual attention (i.e., focused vs. distributed attention) may therefore play a differential role on different attentional processes (alerting, orienting, executive control). In order to address the specificity of cultural and language effects on attention, the present study considered task performances on attentional processes of children whose cultural backgrounds are differently aligned on the Eastern–Western culture continuum and language backgrounds (e.g., monolingual vs. bilingual).

### Attentional Networks Relevant to Culture and Language

#### Three Attentional Processes in the ANT

**FIGURE 1:**
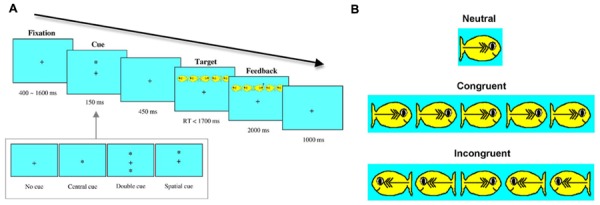
**(A)** The Attention Network Test (ANT) task design, and **(B)** A set of stimuli used in the ANT. Figure retrieved from Rueda et al. (2004).

Three attentional processes have often been considered to serve as a basis for effective attention—the alerting, orienting, and executive control network (Fan et al., 2002). The performance of these attentional networks are critical in allowing us to effectively exercise attentional resources that are involved in processing, organizing, and selectively attending to relevant information (Posner and Fan, 2004), thereby providing success in a variety of complex task demands (Fan et al., 2003). The three attentional networks are accessed by incorporating response time (RT) to cue-target and flanker trials; each trial type are used to measure the alerting and orienting networks, and the executive control network, respectively (Fan et al., 2002). As can be seen in **Figure 1A**, the ANT is a computer generated attention task that begins with a fixation point in the middle of the screen that appears between 400 and 1600 ms, with one of four cues quickly following. The four types of cue-target trials are (1) No Cue (only fixation point appears in the middle), (2) Central Cue (an asterisk appears over the fixation point in the middle of the screen), (3) Double Cue (two asterisks appear on top and on bottom of the fixation point), and (4) Spatial Cue (an asterisk appears either on top or bottom of the fixation point indicating where the target will appear). These four cue-target types are used to compute the performance of the alerting and orienting networks. The performance of the alerting network is examined by the changes in RT resulting from the sensitivity to the presence and absence of cue presentation (i.e., difference in RTs for No Cue – Double Cue trials). This ability fully develops around 10 years of age and beyond (Rueda et al., 2004). The performance of the orienting network is examined by the changes in RT from cues indicating where the target will occur (i.e., difference in RTs for Central Cue – Spatial Cue trials). In the flanker trials (depicted in **Figure 1B**), a participant is instructed to find the target (i.e., yellow *middle* fish) that sometimes appear alone (neutral), or among other surrounding distracter fish that are either facing the same direction (congruent trials) or different direction (incongruent trials.) The performance of the executive control network is examined by the changes in RT from congruent to incongruent trials (i.e., difference in RTs for Incongruent – Congruent trials).

#### Bilingualism and the Executive Control Network

The executive control network measured by the ANT has been related to inhibition, conflict resolution, planning, and cognitive flexibility, and is important for one’s ability to monitor and resolve conflicts in planning, decision-making, error detection, and overcoming habitual actions (Wang and Fan, 2007). Bilinguals have often been found to outperform monolinguals specifically on inhibition and cognitive flexibility, considered to be the result of their frequent exercise for cognitive control when resolving lexical competition (Green, 1998; Bialystok, 1999; Gollan and Kroll, 2001; Bialystok and Martin, 2004; Carlson and Meltzoff, 2008; Kroll et al., 2008; Martin-Rhee and Bialystok, 2008). This cognitive environment plays an important role for the executive control network in both adults (Costa et al., 2008; Pelham and Abrams, 2014) and children (Carlson and Meltzoff, 2008; Yang, unpublished), with stabilization of the executive control network occurring after 7 years of age (Rueda et al., 2004). Despite of the relatively coherent research findings, most research has exclusively been focused on the executive control network given the strong links between bilinguals’ use for inhibition and cognitive flexibility in resolving lexical competition. What is not known, however, is whether bilingualism is solely responsible for the development of the executive control network, or if the interaction between bilingualism *and* culture together may better capture task results on the development of this network.

#### Culture and the Alerting Network

The alerting network has been considered to be responsible for achieving and maintaining *broad sensitivity* to incoming information, where a heightened internal state of arousal is required in preparation for incoming signals (Wang and Fan, 2007). Although research in adults have suggested some bilingual advantage in the alerting network (Costa et al., 2008; Videsott et al., 2012), little to no differences were found in children for this network (Yang, 2007; Kapa and Colombo, 2013; Anton et al., 2014; Yang, unpublished). The present study aims to approach the previous mixed results by focusing on the alerting process in the framework of the Eastern cultural influence. The cultural differences previously mentioned in attentional processing (i.e., Western cultures tend to process individualized information or “analytic” focused attention, while Eastern cultures rely on contextual features to process information or “holistic” distributed attention; Nisbett et al., 2001) may be differentially related to the alerting network. The idea here is that *broad sensitivity* to incoming information in alerting attention may be more influenced by distributed attention processing found in Eastern cultures, where information is broadly processed and attention to objects are *relational and contextualized* to surrounding features (e.g., Masuda and Nisbett, 2001, 2006; Nisbett et al., 2001; Nisbett, 2003; Nisbett and Miyamoto, 2005; Kuwabara and Smith, 2012). Therefore, in order to efficiently process the alerting network, general sensitivity to the presentation of an incoming signal (double cue), or lack thereof (no cue), is vital for success. As such, distributed attention processing frequently adopted in Eastern societies may therefore aid in achieving higher performances in the alerting network and explain why differences were not seen in bilingual children with non-Eastern or non-collectivistic cultural background.

#### Neither Language nor Culture Affect the Performance of the Orienting Network

Orienting visual attention has been defined as disengaging, shifting, and reengaging one’s attention (Posner et al., 1984) and is often considered as the “where” pathway of attention for spatial information processing (Ungerleider and Mishkin, 1982; Wang and Fan, 2007). Spatial information processing has been found to be easier for subjects as the trials are not contingent on cues and stimulus nor target, therefore subjects typically respond faster in the orienting network (Posner, 1980). Moreover, studies on 6- to 10-years old children showed little development for the orienting network overtime, suggesting that areas involved in the orienting network is fully developed in early childhood (Rueda et al., 2004). Due to negligible differences in the development of the orienting network in early childhood (Rueda et al., 2004; Yang, 2007; Yang et al., 2011; Kapa and Colombo, 2013; Anton et al., 2014; Yang, unpublished), little to no language and/or cultural effects are expected on task performances.

In sum, the current hypothesis regarding the attentional networks is that culture may be more relevant for the development of the alerting process, with a particular advantage for Eastern culture due to the distributed attention processing of visual information. Meanwhile, bilingualism may be more influential to the development of the executive control process, compared to other components, due to the implications and frequent use for cognitive control and flexibility among bilingual learners. Finally, the present study hypothesizes that neither language nor culture may be involved for the development of the orienting process, due to previous research demonstrating that it is not contingent on cue and stimulus nor target (Posner, 1980) and the lack of developmental change (Rueda et al., 2004). Therefore, the bilingual advantage in attention may be more relevant in processes that take a longer time to develop in early childhood, such as the alerting and/or executive network.

### Participant Selection: Developmental Issues and the Cultural Continuum

#### Developmental Issues

The idea here is that bilingual individuals are defined not entirely by the types of language one is learning, but also the culture to which they belong. Thus, the interaction between learning multiple languages and coming from multiple cultural backgrounds is a particular area that requires additional parsing to understand the magnitude of the *bilingual* and *cultural* advantage together and on its own. However, there are no systematic studies documenting how and *when* bilingual advantage and the Eastern cultural influence are related Thus, there is little developmental basis for the contribution and collective evidence of language and cultural effects on attentional control. One recent attempt documented that the bilingual advantage effect does not persist throughout development (Tran and Yoshida, 2012). In this study, bilingual children’s attentional control is optimized at 3 years of age, with the effect diminishing at age 5. Further, another attempt suggests that the Eastern cultural influence on executive function tasks emerges early (at 3 years of age), yet its development is rather gradual (Tran et al., under review). These previous studies suggest that the language effect and cultural influence may vary the developmental relation, and that it is important to track broader periods of individuals’ development to observe the effects. Moreover, bilingual advantage and the cultural effect have often been studied across different tasks involving different task difficulty and requiring different levels of language knowledge. This, therefore, simply provides a snapshot of potential relation among these factors and may not fulfill the gap in the current literature. To systematically document and fully compare the influence of language and culture on attention control, 3-years-old children who were at the earliest stage of participating in the ANT (Yoshida et al., 2011) were selected and tested repeatedly until 5 years of age.

#### Cultural Continuum

In order to document the range of potential cultural influences on task results, cultures are presently considered through strict scaling based on the tightness and looseness of societal structure for collectivism and individualism, an indicator that categorizes different cross-cultural dimensions (Hofstede, 1980, 2001, 2003). The participating children resided in three countries: the U.S., Vietnam, and Argentina. The cultural groups were chosen to span Western (U.S.), Western-European with Latin influences (Argentina), and Eastern (Vietnam) cultures that incorporate the proposed continuum from more individualistic to more collectivistic societies (Vuong, 1976; Hofstede, 1980, 2001, 2003; Hui, 1984; Cha, 1994; Ho and Chiu, 1994; Lytle et al., 1995; see **Table 1**). Specifically, backgrounds of Argentinean and Vietnamese learners have seldom been studied in the framework of bilingual and cultural effect on cognitive development (Tang, 2006; Pham and Kohnert, 2008). From these cultural variations, there were two language groups (bilinguals and monolinguals) generated: Monolingual (English, Vietnamese, and Spanish) and bilingual speaking (Vietnamese–English, Vietnamese–Cantonese, and Spanish–English.) The present study included U.S. resident bilingual children whose cultural backgrounds vary. Inclusion of the U.S. children with different cultural backgrounds were analyzed according to their non-U.S. cultural backgrounds, due to studies suggesting the significant influence of the native culture relevant to the study, even in everyday exposure to individualism found in Western cultural practices (Ahadi et al., 1993; Farver and Lee-Shin, 2000; Chao and Tseng, 2002; Parmar et al., 2004; Oh and Lewis, 2008).

**Table 1:** Spectrum of the degrees of collectiveness among different cultures.

Degree of societal structure	Individualistic		Collectivistic
	**Loosely Structured**		**Highly Structured**

Cultural groups	Western	Western-European with Latin Influences	Eastern
Language groups (Country)	English (U.S.)	Spanish (Argentina) and Spanish-English (Argentina/U.S.)	Vietnamese (Vietnam) and Vietnamese–Cantonese (Vietnam/China) and Vietnamese–English (Vietnam/U.S.)

The Institutional Review Board (IRB) in Houston, TX, USA, in collaboration with international school boards at respective districts in Argentina and Vietnam, approved the present study protocols before parental consent were obtained for data collection.

## Materials and Methods

### Participants

Ninety-seven 3-years-old (*M*_age_ = 38.78 months) monolingual and bilingual children from three countries (U.S., Argentina, and Vietnam) participated in the present longitudinal study. Children participated in the ANT for a total of five developmental time points, with each session being 6 months apart (*M*_ages_ = 45.35, 51.20, 57.52, and 63.35 months at Time 2, 3, 4, and 5, respectively). Children were recruited from communities in Houston, TX, USA; Salta, Metán, and San Miguel de Tucumán in Argentina; and Ðồng Nai, Việt Nam. As can be seen in **Table 2**, both monolingual and bilingual children were recruited in the United States and Vietnam, but only monolingual children were recruited in Argentina because it was difficult to recruit bilingual learners with similar bilingual learning environments and SES backgrounds in this context. Specifically, most bilingual children in Argentina learn a second language while in school, making them older (at least 4 years old) and their “bilingual” learning environment less natural than their U.S. and Vietnam peers. Moreover, most bilingual Argentinean children had substantially higher SES background, which has been found to have measureable effects on children’s cognitive task performances (e.g., Mezzacappa, 2004; Noble et al., 2005). All children were attending preschool at the time of testing.

**Table 2:** Sample characteristics.

Time	Language status	*N*	Mean age (range) in months
1	English	14	37.82 (35.56–41.94)
	Spanish	19	38.38 (31.09–46.48)
	Vietnamese	20	38.08 (31.97–42.57)
	Spanish–English	13	39.80 (35.56–45.53)
	Vietnamese–English	15	40.44 (36.18–45.53)
	Vietnamese–Cantonese	16	38.21 (31.18–45.16)
2	English	14	44.41 (41.68–48.42)
	Spanish	19	45.18 (37.76–53.29)
	Vietnamese	20	44.56 (38.45–49.05)
	Spanish–English	13	46.33 (42.73–51.51)
	Vietnamese–English	15	47.01 (42.17–51.55)
	Vietnamese–Cantonese	16	44.43 (36.45–51.05)
3	English	13	51.18 (48.36–55.03)
	Spanish	19	50.21 (42.80–58.32)
	Vietnamese	20	50.53 (44.51–55.20)
	Spanish–English	13	52.28 (48.39–57.96)
	Vietnamese–English	15	53.88 (47.76–59.97)
	Vietnamese–Cantonese	16	49.86 (42.53–56.94)
4	English	11	57.56 (54.08–61.28)
	Spanish	19	55.73 (49.84–64.05)
	Vietnamese	20	55.55 (49.47–60.16)
	Spanish–English	13	58.34 (54.41–63.03)
	Vietnamese–English	11	60.54 (54.87–67.53)
	Vietnamese–Cantonese	8	57.42 (48.45–62.27)
5	English	10	63.44 (58.91–69.18)
	Spanish	19	62.55 (55.36–70.72)
	Vietnamese	19	61.43 (55.49–65.89)
	Spanish–English	13	64.30 (60.40–68.42)
	Vietnamese–English	10	66.71 (61.35–73.13)
	Vietnamese–Cantonese	9	62.56 (54.51–68.32)

The children who participated in the present study were selected to fall within the 50th percentile, middle SES range in the year data was collected (2008–2009), that was defined for each country (based on national statistics) as followed: $50,000 to $74,999 for the U.S. (John and Catherine, 2013), 15.500–21.499 pesos for Argentina (Development Economics LDB database, 2008), and 10,400,000–13,199,999 Dông for Vietnam (Development Economics LDB database, 2008). Furthermore, the middle SES status was also considered in terms of parental education, which has been suggested to play a vital role on chidren’s cognitive task performances and academic achievement (e.g., Smith et al., 1997; Bradley and Corwyn, 2002; Davis-Kean, 2005; Biedinger, 2011). See **Table 3** for more details on the SES scores for each cultural and language groups.

**Table 3:** Socio-economic status (SES) scores.

Country	Culture	Language status	Languages	SES mean scores (SD)	
				Education (out of 20)	Income (out of 9)
Argentina	Western-European with Latin influences	Monolingual	Spanish	13.63 (3.30)	5.00 (2.73)
Vietnam	Eastern	Monolingual	Vietnamese	10.00 (3.28)	7.26 (2.18)
	Eastern	Bilingual	Vietnamese–Cantonese	8.34 (3.24)	5.29 (3.15)
U.S.	Western	Monolingual	English	16.67 (1.92)	7.45 (1.44)
	Western-European with Latin influences	Bilingual	Spanish–English	16.97 (1.49)	7.00 (2.13)
	Eastern	Bilingual	Vietnamese–English	14.31 (3.33)	5.88 (1.72)

### Stimulus Materials

The ANT (Fan et al., 2002; Rueda et al., 2004) was administered using E-Prime software on a 15″ touch-screen laptop computer to measure for selection and RT. The ANT used in the present study is the original child version downloaded from Dr. Jin Fan’s webpage (https://www.sacklerinstitute.org/cornell/assays_and_tools/ant/jin.fan/).

Children’s demographic assessments were conducted using the John and Catherine (2013; MacArthur Network on SES & Health website), a parent questionnaire consisting of 16 questions on SES and the child’s health. We also assessed children’s productive vocabularies in order to provide a rough screening for developmental delays in children across the various language groups. Parents were asked to complete the MacArthur Communicative Developmental Inventory (MCDI, toddler form; Fenson et al., 1993). The English, Chinese, and Spanish checklists were independently developed and normalized (Fenson et al., 1993; Ogura and Watamaki, 1997; also Ogura et al., 1993). Due to the lack of a Vietnamese vocabulary checklist, the Vietnamese version was adapted from the Chinese and Japanese MCDIs, with additional replacements of items native to the Vietnamese culture (i.e., food, drinks, etc.). Parents of bilingual children were asked to fill out two vocabulary checklists that correspond to the languages their child were exposed to. In the present sample, we only included children whose total vocabulary fell above the 20th percentile.

For productive vocabulary scores, we computed the total number of words in their conceptual vocabulary knowledge. For bilinguals, conceptual knowledge was computed on the basis of discounting any overlapping words that exist between the two languages from the total number of concepts known in both languages. Conceptual knowledge is considered a more valid measure of bilinguals’ vocabulary knowledge, especially when comparing with their monolingual counterparts (Umbel et al., 1992; Pearson et al., 1993; Alvarado, 2000; Bialystok, 2001; Oller and Pearson, 2002; Oller, 2005). Analysis comparing conceptual vocabularies across language groups and cultural groups demonstrated no significant differences (*p* > 0.1). See **Table 4**.

**Table 4:** Mean productive vocabulary on the MacArthur–Bates Communicative Development Inventories (MCDI) for Conceptual Knowledge at Time 1 by Language and Cultural Groups.

Conceptual knowledge	Language groups		Cultural groups		
	Monolingual	Bilingual	Western	Western-European	Eastern
Total number of words	287	302	272	302	338

### Procedure

For each visit prior to the children participating in the ANT, parents completed the John and Catherine (2013) MacArthur SES and the MCDI forms. All of the sessions were conducted in a quiet room, where children were instructed to sit on a small chair across from a touchscreen laptop computer. The ANT was administered at all five time points. For bilinguals, the ANT was administered in their dominant language. The dominant language was determined by parental reports on child’s language exposure—number of hours in a day, how many days in a week, with whom, and since what age—for each language.

### Measure

#### Attention Network Test (ANT; Fan et al., 2002; Rueda et al., 2004)

The ANT measures the three attentional networks (alerting, orienting, and executive control) in terms of accuracy and RT.

##### Practice trials

There were a total of 10 practice/familiarization trials. During the practice trials, children were instructed to feed the hungry fish as fast as they can by touching its mouth with their index finger. The target (i.e., hungry fish) is either a single fish (neutral condition) or the middle fish in a row of five fish. The fish could appear above, on, or below the fixation point. The row of five fish could face left or right, and the stimuli could be in a congruent or incongruent direction. The congruent trial will have all five fish facing the same direction (→→→→→ or ←←←←←) and the incongruent trial will display the middle fish facing the opposite direction from the others (→→←→→ or ←←→←←). See **Figure 1B**. Children were told that sometimes the fish would appear alone, and other times it would swim together with other fish. In all cases, they were instructed to concentrate on the middle fish—the hungry fish. They were also asked to keep their eyes on the centered fixation point (+) that is displayed throughout the task. Once they were familiarized with the task, testing trials were administered.

##### Testing trials

A total of 48 trials were presented in two blocks (i.e., 24 trials each block) with a 60 s break in-between the blocks. The procedure was identical to the practice trials, except the experimenter no longer provided feedback. Instead, participants were presented with trials that were accompanied by automated sound feedback: “Woohoo!” for correct responses and a buzzer sound for incorrect responses. See **Figure 1A**. Completion times were ∼10–15 min. The dependent measures, accuracy (proportion correct) and RT (in ms) were recorded for analysis.

## Results

First, we consider language and cultural group differences on *overall* accuracy and *overall* RT on the ANT. This provides an overview regarding task performances among the different groups of children for subsequent analyses. Second, we consider the effects of culture, language status (bilingualism), time, and SES on each child’s ANT task performances. As recommended in the longitudinal data analysis literature (e.g., Singer and Willett, 2003), a series of linear mixed model analyses were performed. For the *overall ANT networks* (all attentional networks in *one* model) and *separate ANT networks* (attentional networks in *different* models) model analysis, the model of best fit was used to compare to an unconditional random intercept model (baseline). Monolingual Western children at time 1 were used as the baseline comparison group. ANOVAs were used to document for general effects, while parameter estimates were analyzed to examine for specific effects of each factor—culture, language status, time, and SES—on task performances. We then consider the magnitude of each factor on task performances over time. All analyses were conducted using the script-based statistical computing software, R (R Core Team, 2013).

### Model Analyses

Following the longitudinal data analysis literature (e.g., Singer and Willett, 2003), linear mixed model analyses were chosen for the present analyses. To test the key hypothesis—whether different attentional processes are uniquely mediated by culture and language status—we first conducted an ANOVA on the *overall ANT networks* model of best fit (one for RT, one for accuracy) that included all attentional networks. For this analysis, the effect of time (1, 2, 3, 4, and 5; random factor), language status (bilingual and monolingual; fixed factor), culture (Western, Western-European with Latin influences, and Eastern; fixed factor), SES (education and income; fixed factor), ANT networks (alerting, orienting, and executive control; fixed factor), time and language status interaction, time and culture interaction, language status and ANT networks interaction, and culture and ANT networks interaction were used to predict the ANT scores. Time was chosen to be a random factor because of the present developmental approach in capturing the relevant period broadly, therefore allowing specific documentations of the relation between culture and language on different attentional networks within the period. The ANT scores were computed using the grand mean of all ANT at time 1, which allows the scores to be regressed for growth modeling (Menard, 2008). This analysis provides an overview regarding the *general* effects of each factor on task performances over time. We then conducted ANOVAs on the *separate ANT network* model of best fit—three attentional networks (alerting, orienting, and executive control) using two types of analysis (accuracy and RT)—to analyze the effects of each factor on *specific* ANT performances.

In order to document the model of best fit, we compared models to an unconditional random intercept model (baseline). For both the overall and separate models, the unconditional random intercept model was centered with monolingual (language status; fixed factor), Western (culture; fixed factor), time 1 (random factor), and SES_min_ (education and income; fixed factors) as the baseline comparison group. We selected a random intercept and random slope model allowing time and intercept to vary across individuals, with all other factors—language status, culture, SES, and interactions among factors—to be fixed. Correlations among individuals were controlled for, allowing slope and intercept to vary.

To evaluate the models of best fit, one goodness-of-fit index was used (e.g., Singer and Willett, 2003): the Akaike’s information criterion (AIC). Models that produced the smallest AIC value were preferred, indicating a goodness of fit that accounts for the variability from the number of estimated parameters included in the model. Finally, to further understand the magnitude of each factor on task performances over time, parameter estimates were examined for each model.

### Overall Performance among Different Language and Cultural Groups

**FIGURE 2:**
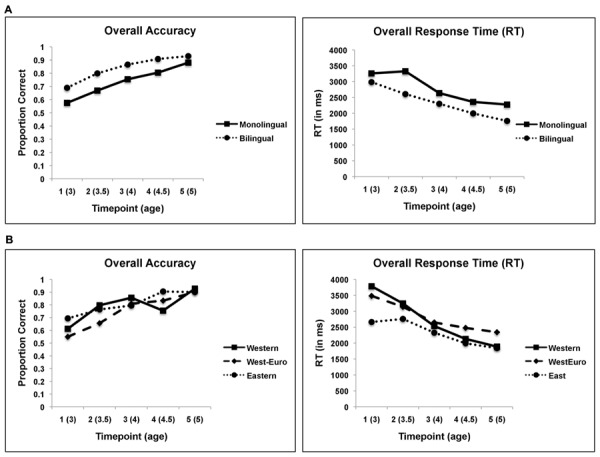
**Overall task performance (accuracy and RT) across all time points by (A) Language Group and by (B) Culture Group**.

Bilinguals performed significantly better than their monolingual peers on accuracy at all time points (*p* < 0.05) and on RT from Time 2 to Time 5 (*p* < 0.05). See **Figure 2A**. *Post hoc* analyses on the overall performance of the different cultural groups further demonstrate that, on average, Eastern children performed significantly faster (*p* < 0.05) and are more accurate than the Western-European with Latin influences children at Time 1, 3, 4, and 5. Performance between Eastern and Western children, however, were comparable across time. See **Figure 2B**.

### General Effect with Overall Network Accuracy and RT Model

The present results reveal that although there are some related developmental changes for RT, bilingual advantage on overall accuracy persists across development. This sets up the stage for the goal of the present study—understanding the relation between culture and language on different attentional networks.

#### Overall Network RT Model

Analyses conducted via an ANOVA on the overall *network RT* ANT model revealed a significant culture and ANT networks interaction [*F*(6,857) = 2.04, *p* < 0.05], suggesting that culture plays a differing role in the RT of specific attentional networks. Furthermore, a main interaction of ANT networks [*F*(3,857) = 4.84, *p* < 0.001] demonstrates that each network has a different developmental trajectory, supporting previous works by Rueda et al. (2004). The magnitude of the differences was further examined by parameter estimates that were standardized by taking the difference of the performance scores of each individual from the grand mean of the ANT network RT at time 1. Scores were then calculated over the SD of performance score of each individual (with a maximum effect size of 2000 ms). That is, score differences were divided by the SD for all participants.

**FIGURE 3:**
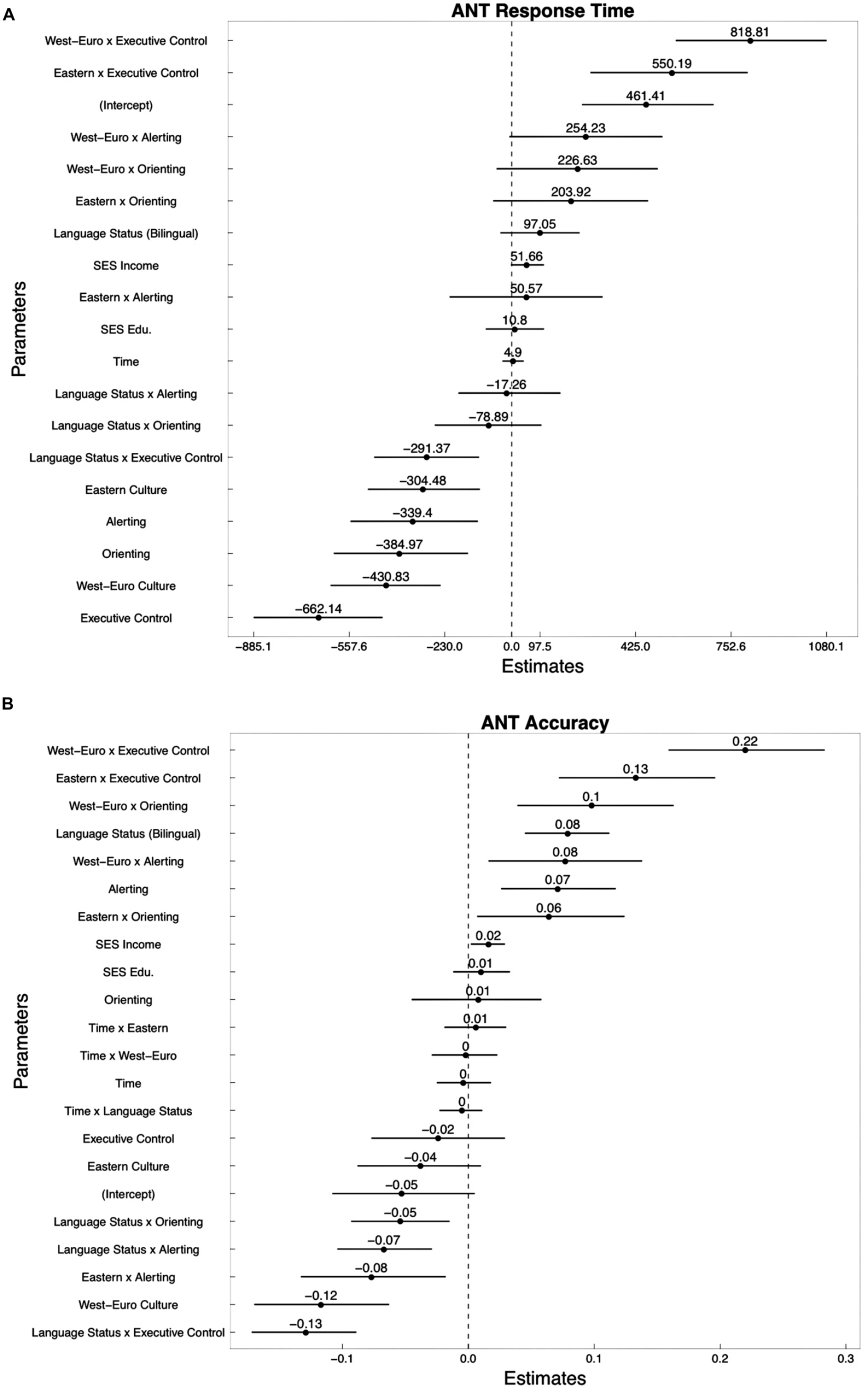
**Coefficient (parameter estimates) plots for the model of best fit on the ANT for (A) Response Time and (B) Accuracy**.

Parameter estimates for the baseline comparison group (intercept) indicate that at time 1, monolingual Western children performed on average a score of 461.41 ms on the ANT task (*p* < 0.01). However, the largest effect size was seen in children from Western-European with Latin influences and Eastern culture. As can be seen in **Figure 3A**, when all other factors are controlled, parameter estimate comparisons reveal that performance on the executive control network is largely influenced by the Western-European with Latin influences (829.67 out of 1500, SE = 266.16; *p* < 0.001) and Eastern (556.05 out of 1500, SE = 256.18; *p* < 0.01) culture. This indicates that monolingual Spanish in Argentina, bilingual Spanish-English and Vietnamese-English in the U.S., and bilingual Vietnamese–Cantonese and monolingual Vietnamese children in Vietnam performed better than their Western counterparts in the U.S. Moreover, performance of children from the Eastern and Western-European with Latin influences culture suggests that they were more “efficient” (smaller difference between conflicting information, determined by values closer to 0; Fan et al., 2002) and relatively stable across time. There were no systematic differences between language groups on the RT of different attentional networks.

#### Overall Network Accuracy Model

Analyses on the overall *network accuracy* ANT model revealed a significant culture and ANT networks interaction [*F*(6,873) = 5.12, *p* < 0.001], suggesting that culture plays a differing role in the accuracy of specific attentional networks (see **Figure 3B**). Moreover, a significant interaction between language and ANT networks was found, [*F*(3,873) = 3.56, *p* < 0.01], suggesting that monolingual and bilinguals perform differently on different networks. For the baseline comparison group (intercept), parameter estimates indicate that monolingual Western children initially performed slightly below average on the ANT task (*p* < 0.01). However, similar to the results for the *network RT* ANT model, the largest effect size was seen in children from the Western-European with Latin influences and Eastern culture. Here, scores are calculated over the SD of performance score for each individual and considered in terms of proportion (with a maximum effect size of 1.0). As can be seen in **Figure 3B**, performance on the executive control network is largely influenced by the Western-European with Latin influences (0.22 out of 1.0, SE = 0.06; *p* < 0.001) and Eastern (0.13 out of 1.0, SE = 0.06; *p* < 0.01) culture. This indicates that monolingual Spanish in Argentina, bilingual Spanish–English and Vietnamese–English in the U.S., and bilingual Vietnamese–Cantonese and monolingual Vietnamese children in Vietnam performed better than their Western counterparts in the U.S.

### Specific Effects with Separate Network Models

From the general analyses on the overall models, results demonstrate a significant main effect of attentional networks for accuracy and for model, *F*(3,857) = 4.84, *p* < 0.001, *F*(3,873) = 3.56, *p* < 0.01, respectively. These results demonstrates that each network (i.e., altering, orienting, and executive control) functions differently from each other, as supported by previous work citing independencies of individual attentional networks (Fan et al., 2002; Posner and Fan, 2004; Rueda et al., 2004) that involves different neural areas (Wang and Fan, 2007). However, to further understand the interaction between culture and networks and language and networks, individual attentional networks were analyzed separately. ANOVAs were conducted on the model of best fit on accuracy and RT for each respective attentional network. See **Table 5** for full specifications of the parameter estimates included for the model of best fit for each individual network models.

**Table 5:** Parameter estimates for individual network models (Accuracy and RT).

(A) Accuracy				
	**Networks RT**								
	
	**Alerting**			**Orienting**			**Executive Control**		
	**Estimates**	**SE**	****Pr*(**>**|*t*|)***	**Estimates**	**SE**	****Pr*(**>**|*t*|)***	**Estimates**	**SE**	****Pr*(**>**|*t*|)***

Time	248.05	211.58	n.s.	11.99	177.24	n.s.	-4.99	87.52	n.s.
Language Status	250.20	251.28	n.s.	-57.83	202.71	n.s.	-196.34	210.60	n.s.
Eastern	-190.93	362.78	n.s.	30.37	292.51	n.s.	173.22	185.83	n.s.
West-Euro	-127.02	376.52	n.s.	-137.74	303.72	n.s.	319.64	157.64	0.01
SES (Education)	—	—	—	—	—	—	-61.97	158.59	n.s.
SES (Income)	-65.24	135.34	n.s.	226.76	107.37	0.01	-186.90	94.63	0.05
Time × Language status	-159.62	174.48	n.s.	81.22	146.11	n.s.	4.89	140.75	n.s.
Time × Eastern	-99.56	253.75	n.s.	-104.41	212.64	n.s.	—	—	—
Time × West-Euro	-104.28	261.15	n.s.	-4.71	218.72	n.s.	—	—	—

**(B) Response time**				

	**Networks Accuracy**								
	
	**Alerting**			**Orienting**			**Executive Control**		
	**Estimates**	**SE**	***Pr(>| t|)***	**Estimates**	**SE**	***Pr(>| t|)***	**Estimates**	**SE**	***Pr(>| t|)***

Time	-0.02	0.04	n.s.	-0.01	0.05	n.s.	-0.01	0.02	n.s.
Language status	0.03	0.05	n.s.	0.05	0.06	n.s.	-0.02	0.04	n.s.
Eastern	-0.19	0.07	0.01	0.02	0.08	n.s.	0.08	0.05	0.05
West-Euro	-0.10	0.07	n.s.	0.01	0.09	n.s.	0.09	0.04	0.01
SES (Education)	-0.04	0.04	n.s.	—	—	—	-0.02	0.04	n.s.
SES (Income)	0.01	0.02	n.s.	0.03	0.03	n.s.	-0.01	0.02	n.s.
Time × Language status.	-0.02	0.04	n.s.	-0.02	0.04	n.s.	-0.03	0.03	n.s.
Time × Eastern	0.04	0.05	n.s.	0.01	0.06	n.s.	—	—	—
Time × West-Euro	0.04	0.05	n.s.	-0.03	0.06	n.s.	—	—	—

*Centering (average distance to center) for Intercept based on Language (monolingual), Culture (Western), Time (1), and *SES*_min_ (education: 0.05, income: 0.11) as baseline. —: not included in model of best fit*.

#### Alerting Network

Analyses conducted on accuracy for the alerting network revealed a significant main effect of culture on task performances, [*F*(2,81) = 3.69, *p* < 0.01], demonstrating the influence of culture on the development of the alerting network. This supports the hypothesis regarding the influence of culture on the development of the alerting network. There were no significant main effects of culture nor language for RT on the alerting network.

#### Orienting Network

The analyses conducted on accuracy and RT for the orienting network revealed no significant differences between culture and language. The results support the hypothesis regarding the least relevancy of individuals’ culture and language background to the development of the orienting network.

#### Executive Control Network

Lastly, ANOVAs conducted on the executive control network for accuracy revealed a significant main effect of culture on task performances, [*F*(2,147) = 2.49, *p* < 0.05]. This demonstrates the role of culture on the development of the executive control network. Analyses conducted on RT, however, revealed no significant effects of language nor culture on the executive control network.

## Discussion

Despite significant findings on the relation between bilingualism and attentional processes, most of the existing work suggesting early cognitive advantages for bilinguals has taken place in the United States, Canada, and/or other English-speaking countries. In an effort to reflect the complexity among bilingual individuals’ cultural backgrounds and recognizing the potential role of culture in the bilingual advantage, researchers have examined the effects of *language* background, such as bilingualism, (i.e., Carlson and Meltzoff, 2008) or *culture* (Oh and Lewis, 2008) on various types of tasks measuring cognitive control. The present study had two related aims. First, the present study was designed to document the relation between bilingualism and cultural influence developmentally. To do this, the present study conducted systematic comparisons considering how these factors together or alone relates to early attentional control. Bilingual studies concerning the cultural effect typically include immigrant children, thereby confounding the true nature of the bilingual effect on early cognitive advantages. Immigration, alone, defines one’s cultural beliefs, attitudes, norms, values, and plays an imminent role in different cognitive systems (e.g., Bornstein et al., 1991; Sigel and Kim, 1996; el Moussaoui and Braster, 2011). Recent studies suggest that family and cultural values (e.g., Sabbagh et al., 2006; Oh and Lewis, 2008; Bialystok and Viswanathan, 2009), SES (Carlson and Meltzoff, 2008; Calvo and Bialystok, 2009), and language learning experiences (e.g., Bialystok, 2001, 2009; Carlson and Meltzoff, 2008; Yoshida et al., 2011; Yang, unpublished) might be important factors to consider when assessing bilingual children’s cognitive skills. Second, the present study focuses on specific components of attention to understand *how* the bilingual advantage and cultural effect are similarly and differentially generated through the ANT components (e.g., Costa et al., 2009; Kapa and Colombo, 2013; Anton et al., 2014; Pelham and Abrams, 2014).

Given the systematic comparisons, current results demonstrate that individual’s cultural background plays a vital role in the development of the alerting and executive control networks, which have also been reported as part of the bilingual advantage (e.g., Carlson and Meltzoff, 2008; Costa et al., 2008; Videsott et al., 2012; Marzecová et al., 2013; Pelham and Abrams, 2014; Yang, unpublished). This suggests that there are potential overlap between bilingualism and cultural influences, and conflicting reports between existing bilingual advantages might be due in part to the individual participant’s degree of different cultural influences (e.g., Costa et al., 2009; Yang et al., 2011; Kapa and Colombo, 2013; Yang, unpublished) and lack thereof (Anton et al., 2014; Pelham and Abrams, 2014). For example, when cultural background is defined more narrowly or controlled across individuals, bilingual advantage persists (Costa et al., 2008; Yang et al., 2011). In both studies, one in Korea and one in Spain, a positive bilingual effect on executive attentional control persists when comparing monolinguals to bilingual children (Yang et al., 2011) and adults (Costa et al., 2008). Moreover, Korean and Spanish participants in the reported studies are ranked low on the Individualism Index Values scale, a cultural measure for individualism–collectivism as proposed by Hofstede (2001), where South Korea and Spain are considered collectivistic societies (Hofstede, 1980, 2001, 2003; Cha, 1994; Oh and Lewis, 2008). This also suggests that the bilingual advantages documented here appear to override the cultural effect, yet there might be potential additive effect in bilingualism (e.g., Ianco-Worrall, 1972; Hakuta and Bialystok, 1994; Cook, 1997; Adescope et al., 2010). In this case, bilingual advantage is not only present, but possibly stronger than the bilingual advantages found in studies comparing bilinguals and monolinguals within highly individualistic cultures. This raises the possibility of the effect itself being graded in nature, rather than all or none.

As a whole, the present results suggest that characteristics inherent in collectivistic societies may play an important role on the alerting and executive control network. These networks are particularly relevant for maintaining broad sensitivity to incoming information and resolving conflicting cues presented. This seems to fit to Eastern culture emphasizing broader attention in visual tasks, but also behavioral practices for self-control (Hofstede, 1980, 2003; Hui, 1984; Cha, 1994; Ho and Chiu, 1994; Lytle et al., 1995; Oh and Lewis, 2008). Further, the differential patterns of attentional processing demonstrated in Eastern cultures could also be partially due to the behavioral differences exhibited during testing. For example, children with the Eastern influence may listen to the instruction more carefully, better at sitting still, etc. given their cultural practices on obedience to authority figures, demoting play, advanced academic training, and early self-control (e.g., Tobin et al., 1989; Ho, 1994; Wu, 1996; Chen et al., 1998; Nisbett et al., 2001; Parmar et al., 2004; Oh and Lewis, 2008). This is an important question regarding the nature of cultural influences on the development of attentional control. Finally, the lack of cultural and bilingual effect on the orienting network suggests that the effect may be sensitive only in complex attentional situations, such as information processing and maintenance, inhibition, and cognitive flexibility of cues and target stimuli. This further adds to previous research regarding negligible developmental differences found in the orienting network. Thus, attentional processes that take longer to develop may be more sensitive to language and cultural factors.

Culture, here, is just the tip of the iceberg in explaining some of the possible discrepancies in current literature on bilingual cognitive advantage. Other factors such as simultaneous vs. sequential learning and/or unbalanced vs. balancedness between languages in bilingualism may be important factors to consider when accessing bilingual cognitive advantage. The present study suggests that neither language nor culture alone can account for the cognitive advantages demonstrated in children, but rather interactions between possible factors such as language and culture must be taken into consideration when considering bilinguals’ development of attentional control. And finally, how each factor may be uniquely relevant to specific processes of attention, thereby changing the relation in the course of the rapidly changing development. The present study recognizes that language and culture are uniquely bound and plays an influential role on learning and support for complex learning situations among children.

## Conflict of Interest Statement

The Guest Associate Editor Eliana Colunga declares that, despite having collaborated on the Research Topic (Context Specific Nature of Bilingual Cognitive Advantages) with author Hanako Yoshida, the review process was handled objectively. The authors declare that the research was conducted in the absence of any commercial or financial relationships that could be construed as a potential conflict of interest.
